# Convergence in the Bilingual Lexicon: A Pre-registered Replication of Previous Studies

**DOI:** 10.3389/fpsyg.2016.02081

**Published:** 2017-01-23

**Authors:** Anne White, Barbara C. Malt, Gert Storms

**Affiliations:** ^1^Department of Experimental Psychology, KU LeuvenLeuven, Belgium; ^2^Department of Psychology, Lehigh University, BethlehemPA, USA

**Keywords:** bilingualism, bilingual lexicon, lexical organization, semantic convergence, naming

## Abstract

Naming patterns of bilinguals have been found to converge and form a new intermediate language system from elements of both the bilinguals’ languages. This converged naming pattern differs from the monolingual naming patterns of both a bilingual’s languages. We conducted a pre-registered replication study of experiments addressing the question whether there is a convergence between a bilingual’s both lexicons. The replication used an enlarged set of stimuli of common household containers, providing generalizability, and more reliable representations of the semantic domain. Both an analysis at the group-level and at the individual level of the correlations between naming patterns reject the two-pattern hypothesis that poses that bilinguals use two monolingual-like naming patterns, one for each of their two languages. However, the results of the original study and the replication comply with the one-pattern hypothesis, which poses that bilinguals converge the naming patterns of their two languages and form a compromise. Since this convergence is only partial the naming pattern in bilinguals corresponds to a moderate version of the one-pattern hypothesis. These findings are further confirmed by a representation of the semantic domain in a multidimensional space and the finding of shorter distances between bilingual category centers than monolingual category centers in this multidimensional space both in the original and in the replication study.

## Introduction

Currently the field of psychological research suffers from a replication crisis (Pashler and Wagenmakers, 2012). The Open Science Collaboration (2015) published a large scale replication effort of 100 experiments in which only 36% of the replications proved to be statistically significant compared to 97% of the original studies. Reproducibility should be one of the core principles of science, and replication studies are a way to assess and improve reproducibility. A successful replication adds to the evidence of the credibility of a particular study and provides support for the earlier obtained results. Replication helps control for sampling errors, artifacts, or even fraud (Schmidt, 2009). A single study offers tentative evidence whereas a successful replication offers confirmatory evidence (Open Science Collaboration, 2015). Transparency and openness are also acknowledged as core principles of conducting science (Miguel et al., 2014; Nosek et al., 2015). Preregistration of the study together with analysis plans is an important tool to create transparency between exploratory and confirmatory research (Bakker et al., 2012). In this paper, we present a pre-registered replication study of convergence in the bilingual lexicon, a phenomenon in which words in the two languages of bilinguals are more alike in meaning and use, as compared to the monolingual word meaning and use in those same languages.

Broadly speaking, bilingual convergence describes increased similarity between some elements of a bilingual’s two languages. (Used in this sense, convergence refers to the outcome of a developmental trajectory and not the process of converging.) Convergence enhances already existing similarities between those languages (Ameel et al., 2009; Alferink and Gullberg, 2013). This increased similarity manifests itself at different language levels including phonology and phonetics (Bullock, 2004; Chang, 2013) and morphology and syntax (Kantola and van Gompel, 2011; Sanchez, 2012; Bernolet et al., 2013).

**FIGURE 1:**
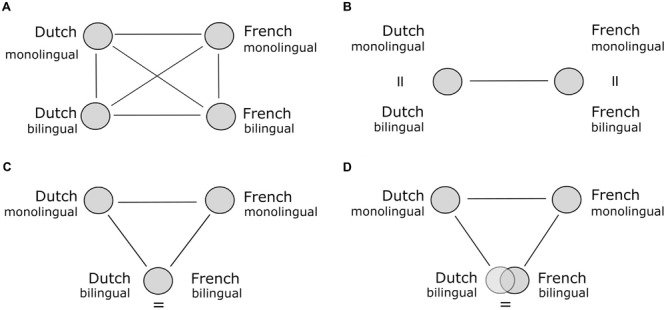
**Schematic representations of the different hypotheses regarding the bilingual lexicon, adopted from Ameel et al. (2005), the circles representing naming patterns and the lines the correlations.** The left upper panel **(A)** represents the four naming patterns and six correlations assessed. The remaining charts show the pattern of correlations corresponding with the two-pattern hypothesis **(B)**, the strong version of the one-pattern hypothesis **(C)** and the moderate version of the one-pattern hypothesis **(D)**.

Differences between sound systems and grammars of languages are readily apparent, making it obvious that bilinguals must somehow navigate these differences in acquiring the two languages. Also the words of the languages (both their meanings and patterns of use) may differ in subtle ways for many domains, such as color (Kay et al., 1997; Roberson and Davidoff, 2000; Regier et al., 2007), motion and movement (Talmy, 1985; Slobin, 1996), and emotion (Wierzbicka, 1999) among others. Differences in the way how languages map words onto referents exist even for common concrete words for familiar objects (e.g., Malt et al., 1999; Pavlenko, 2009; see Malt and Majid, 2013 for review). This implies that bilinguals must either develop and maintain two separate, monolingual-like naming patterns in their two languages or in some way develop a more shared semantic system. Ameel et al. (2005) termed the first possibility the two-pattern hypothesis (see **Figure 1B**). This hypothesis predicts that the naming patterns of both a bilingual’s languages will be identical to the monolingual naming patterns of these languages. In contrast, the strong one-pattern hypothesis suggests that bilinguals fully merge their naming patterns. In this completely converged naming pattern, the patterns in the two languages are identical to each other (**Figure 1C**). Ameel et al. (2005) tested these hypotheses by asking functionally monolingual Belgian speakers of French and of Dutch and Belgian bilingual speakers of both languages to name pictures of household containers and dishwares. They found that the bilinguals’ naming patterns in the two languages were more similar than were those of the monolinguals of each language. This finding is consistent with the one-pattern hypothesis. However, the convergence was only partial, pointing to a more moderate version of the one-pattern hypothesis (**Figure 1D**). The initial model with six correlations between each pair of language groups is represented in **Figure 1A**.

Ameel et al. (2009) provided additional support for partially merged bilingual semantics by examining the representations in a multidimensional semantic space. They found convergence in both lexical category centers and boundaries. Convergence has now been shown in the lexicons of both early (Ameel et al., 2005, 2009) and late bilinguals (Zinszer et al., 2014; Malt et al., 2015), and in a forced choice task (Malt and Lebkuecher, 2016) as well as the more usual free naming paradigm. It also manifests itself over the course of bilingual language development: Storms et al. (2015) found convergence at all ages from age 5 onwards with a steadily increasing correlation between the bilinguals’ both languages with age, suggesting a stronger convergence with age.

In the current study, we replicated the work of Ameel et al. (2005) and an additional analysis of Ameel et al. (2009) on the same dataset, using a larger set of stimuli. We expanded the original set consisting of 73 pictures of containers with an additional 119 pictures. One benefit of the expansion is that studies providing evidence for convergence in the bilingual lexicon for the most part draw on the same original stimulus set (Ameel et al., 2005; Zinszer et al., 2014; Malt et al., 2015; Storms et al., 2015; Malt and Lebkuecher, 2016) or provide only additional analyses of a dataset from those stimuli (Ameel et al., 2009). A replication with a larger set of stimuli can validate previous findings and enable us to investigate the generalizability of those findings. Second, the new stimulus set contains more exemplars of the main lexical categories leading to a more reliable, denser representation of the lexical domain. The increased density allows for a more detailed comparison between boundaries of the lexical categories of bilinguals versus monolinguals. On the one hand, some previously small categories, like the French and Dutch tube (similar to English tube for toothpaste) were expanded, on the other hand, some smaller categories were added (i.e., Dutch bidon, similar to English jug).

Schmidt (2009) distinguishes two kinds of replication. Direct replication reproduces the original procedure as exactly as possible, whereas conceptual replication uses a method deviating from the original study to test the same hypothesis. Given the large stimulus set we used, several changes were made to the data collection procedure. Therefore, our study can be considered a conceptual replication. Replicability of an effect is mostly dependent on the robustness and the stability of the effect (Bakker et al., 2012; Klein et al., 2014). Conceptual replications of an effect are informative about the robustness of the effect and also about the generalizability to a broader context. Detailed of the deviations from the original study and justifications are described in the “Materials and Methods” section.

## Materials and Methods

Before the start of the data collection this replication study was pre-registered on Open Science Framework (osf.io/cnm3w; Spies et al., 2012). The replication protocol proposed by the Open Science Collaboration included the following elements: selecting the study and key effects from the available articles, contacting the original authors for study materials, preparing a study protocol with analysis plan, obtaining review of the protocol by the original authors and other members, registering the protocol publicly, conducting the replication, writing the final report, and auditing the process and analysis for quality control (Open Science Collaboration, 2015).

This replication study focusses on the findings regarding convergence of bilingual naming patterns reported in Ameel et al. (2005), and one analysis giving additional support reported in Ameel et al. (2009). We opted to focus on replication of the manifestation of convergence itself, leaving findings regarding the nature of this convergence and the complexity of category structure aside. To avoid an excessively long paper, we delimit the topic to whether there is a convergence; thus, the remaining experiments using linear separability and outlier analysis (Ameel et al., 2009) will not be discussed here. Concerning the study materials, we used the original stimulus set expanded with similar stimuli. In the naming experiments, the same instructions of Ameel et al. (2005, 2009) were used. The questionnaire used to assess the language background of the participants was adopted from the original study. Additional sorting data was collected to examine convergence in a geometrical space. With the sorting data a common underlying representation was constructed reflecting the similarity between the objects. A study protocol with analysis plan was drafted, reviewed by the co-authors, and pre-registered on Open Science Framework.

### Participants

#### Naming

We collected naming data of 32 largely monolingual Dutch speaking and 30 largely monolingual French speaking adults. The Dutch speaking participants were students at the Psychology Department of the Katholieke Universiteit Leuven, and received course credits for participation. The monolingual French speaking participants were all students at the Université Catholique de Louvain and were paid for their participation. Similar to the original study, the monolingual participants had some knowledge of the other language through formal instruction at school but they did not use this language in daily activities (with the exception of two French speaking participants and two Dutch speaking participants who indicated an occasional use of the other language). None of the Dutch speaking participants considered themselves fluent in French. Of the French speaking participants, one indicated a high degree of proficiency in Dutch^1^ (through formal instruction), but not at the level of a native speaker.

The 30 bilingual participants were recruited via social media and were paid for their participation. Inclusion criteria are based on age, context, and manner of acquisition of French and Dutch. In this replication, like in the original study, we recruited early simultaneous bilingual participants who were raised by a Dutch speaking mother and French speaking father or vice versa. Both parents consistently spoke their own mother tongue in raising their children from birth onwards. Late bilinguals were not included in this study. Sixteen participants had a French speaking father and Dutch speaking mother, and 14 had the converse. Information concerning language background (proficiency estimates, contact with other languages, etc.) was collected using a language history questionnaire (also used in the original study). This questionnaire addressed the following questions: age, gender, place where the participant was raised, mother tongue of the parents, language spoken with the mother, language spoken with the father, how consistently the same language is spoken with the same parent, what language was spoken in every stage of the school career and during leisure activities, which language is used the most, in which language the participant thinks spontaneously and an estimate of proficiency in both languages. Similar information was gathered from the monolingual participants.

Participants estimated proficiency on a scale from 1 (“not at all proficient: you can barely speak the language”) to 7 (“very proficient: you can speak the language like a native speaker”) for each language. We used the proficiency estimate also used by Ameel et al. (2005) for reasons of comparability. Moreover, this type of self-report measures have been shown to correspond well with performance measures of proficiency such as reaction times (Dufour and Kroll, 1995; Kroll et al., 2002). The mean French proficiency estimate of the Dutch monolinguals was 2.88 (*SD* 0.98), and the mean Dutch proficiency estimate of the French monolinguals was 1.80 (*SD* 1.32). The mean estimates for the bilingual participants were 6.35 (*SD* 0.84) for Dutch and 5.52 (*SD* 0.84) for French. Eight out of 30 participants indicated an equal degree of proficiency in both languages and are therefore considered balanced bilinguals. Of the remaining 22 participants, 19 indicate a higher level of proficiency in Dutch and three participants indicated a higher level of proficiency in French. As a group, the bilingual participants indicated a significantly higher degree of proficiency in Dutch than French, *t*(29) = 3.40, *p* < 0.01. The bilinguals also indicated a significantly lower degree of proficiency in both languages as compared to monolingual participants, *t*(39.18) = -2.96, *p* < 0.01 and *t*(41.78) = -8.17, *p* < 0.001, for Dutch and French, respectively. The characteristics of the participants in the original study are very comparable. The bilingual participants consisted of 25 people with a Dutch-speaking father and a French-speaking mother (14 out of 25) or vice versa (11 out of 25). The mean proficiency estimates in their two languages were also very high: 5.7 for French (*SD* 0.64) and 6.5 for Dutch (*SD* 0.74). The estimates for the non-native language of the monolinguals were 2.8 (*SD* 0.83) for the French competence of Dutch speaking monolinguals and 1.3 (*SD* 0.65) for the Dutch competence of the French-speaking monolinguals.

#### Sorting

Sixty-five other monolingual Dutch adults participated in a sorting task. This task was performed in order to obtain pairwise similarity data. The Dutch speaking participants were students at the Psychology Department of the KU Leuven, and received course credits for participation.

This study was carried out in accordance with the recommendations of the KU Leuven Social and Societal Ethics Committee with written informed consent from all subjects.

### Materials

**FIGURE 2:**
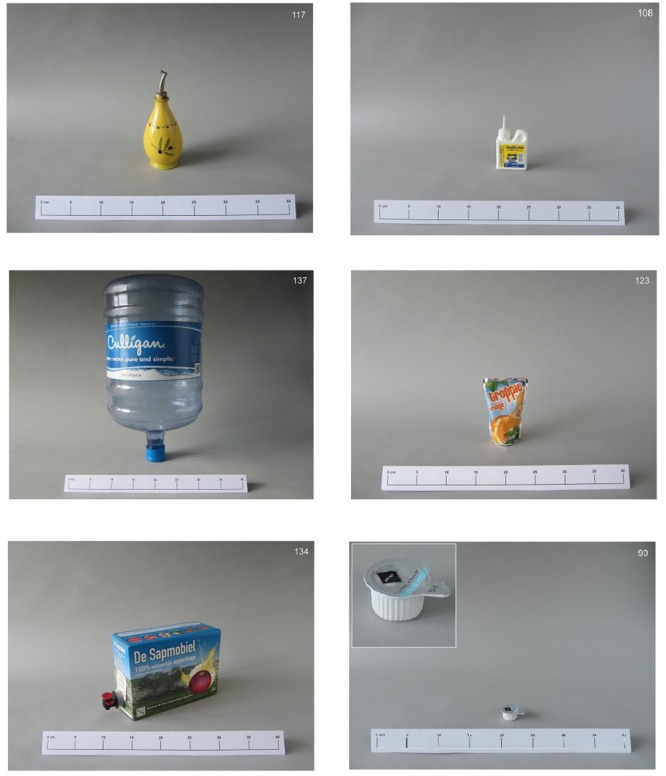
**Some of the new exemplars added to the stimulus set**.

Part of the stimuli used were described in the original study by Ameel et al. (2009, 2005). The original set consisted of 73 pictures of storage containers, referred there to as “the bottles set”; we refer to them here as “containers” for greater accuracy. We expanded the existing set with 119 new stimuli, the full set totaling 192 pictures. This set aims to represent the full range of objects that exist within the domain of household containers. We introduced a larger variety by adding objects of different materials, shapes, and functionalities, and larger and smaller objects. The new pictures were made according to the same guidelines used by Ameel et al. (2005, 2009). The objects were photographed in color against a neutral background with a constant camera distance to preserve relative size. A ruler was included in front of each object to provide additional size information. For objects that were small and hard to inspect on the picture, enlargements in the left upper corner were added, to ensure that the object would be clearly visible despite the small size. Like in the original study, the stimulus set contains objects that can be found at work or at home. The objects present in the set were likely to receive the names bottle, jar, container, or box in English. In Dutch, we anticipated that the most frequently given names would likely be *fles, pot, doos*, and *bus*. In French the most likely names would be *bouteille* or *flacon, pot* and *boîte*. Some examples of the expanded stimulus set can be found in **Figure 2**.

### Procedure

#### Naming

The naming data were collected in an online survey using Qualtrics (Qualtrics, Provo, UT, USA). Participants were asked to name each pictured object. Monolinguals performed the naming task once, bilinguals twice, that is, once in each language. Instructions were identical to those used by Ameel et al. (2005, 2009). Participants were asked to give the objects in the photographs whatever name seemed best or most natural. The participants were explicitly instructed to name the object itself and not what it contains. To prevent order effects, the order of the stimuli was randomized for each participant. The pictures were shown one by one, with room underneath each picture to fill in the appropriate name. Above every picture, a short instruction reminded participants to name the object itself and not the content. In bilinguals the order of languages was counterbalanced, with half first completing the Dutch task first followed by French, half doing the reverse.

#### Sorting

Following Ameel et al. (2005) the data from the sorting task were used to obtain a measure of similarity for each pair of objects since pairwise similarity ratings were not possible given the large number of objects (192). Performing the task on a computer would prevent a good overview of the complete stimulus set. Therefore, we opted to work with a picture set of the objects. Before the start of the sorting task all pictures were spread out over a U-shaped surface, to make sure that participants had a good overview over all objects in the stimulus set. Participants were instructed to first look through the pictures and then place them into piles based on overall qualities of each object, that is, focusing on any feature (or combination of features) of the object that seemed important or natural. Participants were explicitly instructed not to sort two objects into the same pile because of what they contain (such as ketchup and mayonnaise), unless the objects themselves were alike in an overall way. Participants could use as many piles as they wanted and the only restrictions were that participants were not allowed to make less than two piles or to make piles consisting of only one object. Since the stimulus set was considerably large, we adapted the task allowing participants to sort in different levels. They were allowed to organize piles into clusters. To achieve this, participants were informed that they could organize piles into groups consisting of different subgroups and overlap between groups was possible. For example, one was allowed to make piles of “plastic bottle like objects” and “glass bottle like objects” and group them together on a higher level. This was done to obtain more detailed information concerning perceived similarity. The data were recorded by noting down all stimulus numbers in every pile. Pairwise similarity was derived by counting for each of the 18,336 pairs of objects how many participants placed that pair of objects in the same pile. A large number of participants placing the two objects in a pile can be taken to indicate high perceived similarity and a smaller number as indicating lower perceived similarity. Using the split-half technique followed by the Spearman–Brown formula, we first estimated the reliability of the pairwise similarity measure based on the sorting data. Since we aimed at a reliability of at least 0.90, we added participants until this aim was reached as specified in the pre-registered protocol.

### Known Differences with Original Studies

The most important difference in comparison to the original studies by Ameel et al. (2005, 2008, 2009) is the expanded set of stimuli. Concerning the collection of the naming data for the adults, we opted for an online naming task for practical reasons. In the original study, two different sets of stimuli were used (“bottles” set and “dishes” set). Since we expanded the stimulus set, it was not feasible to replicate the study for both sets of stimuli. As to the sorting task, we made the decision to only collect sorting data from monolingual participants. Given that perceived similarity collected from different language groups shows negligible differences, previous studies support the assumption of a common underlying object representation (Malt et al., 1999; Ameel et al., 2005). People of different languages and cultures share a perception of the similarity among entities within at least some domains (e.g., common household containers: Malt et al., 1999; Ameel et al., 2005; color: Roberson and Davidoff, 2000; human locomotion: Malt et al., 2014; and spatial relations: Munnich et al., 2001). As mentioned above, we also allowed the participants to sort in different levels in order to obtain more detailed information concerning perceived similarity (see earlier). In the original study, participants completed both the naming task and the sorting task. Given the large dataset, and the longer duration of the testing procedure, we opted to gather the sorting data with participants who did not do the naming task.

## Results

### Naming Patterns for the Bottles Set for Dutch- and French-Speaking Monolinguals

The study of Ameel et al. (2005) first demonstrated that monolingual Dutch and French speaking adults display different naming patterns despite a shared perception of similarity between objects, replicating the findings of Malt et al. (1999). We focused on the studies regarding bilingualism and not the finding of cross-linguistic differences in naming patterns. After all, there is extensive evidence in literature that different languages demonstrate different naming patterns. Various studies in addition to those of Malt et al. (1999) and Ameel et al. (2005) show evidence for cross-linguistic differences in how people carve up the world around us (see Malt and Wolff, 2010, for an overview).

In order to analyze the naming data, we first tallied the frequency of each name produced for each object. Following the method described by Ameel et al. (2005, 2009) tallies were based on the head noun of the response (that is, adjectives were not taken into account) and diminutive forms of names were combined with the non-diminutive forms into a single category^2^. Naming patterns were studied by analyzing dominant names and calculating the name distribution. The dominant name of a stimulus is the name that is given most frequently for each object. The categories *fles, pot*, and *bus* together encompass 64% of the stimulus set. These categories encompassed 74% of the stimulus set of Ameel et al. (2005). If we take into account a new main category *doos* appearing in the replication data, the four main categories encompass 72% of the data, compared to 79% in the data of Ameel et al. (2005). Supplementary Table 1 presenting the linguistic categories for Dutch and French monolinguals determined based on the dominant names.

However, when taking into account dominant names only, valuable information in the data is lost since only a small number of the objects were named unanimously by the participants. Only 8 and 6 out of 192 objects were called by one single name by every participant in Dutch and French, respectively. The name distribution is an object × names matrix, in which every number indicates the number of times a particular name was produced for a particular object. This name distribution matrix is used to create a name similarity matrix (object × object) by correlating the name distribution of an object with the name distribution of every other object. To this end a Pearson correlation was calculated for every one of the (192 × [192 - 1])/2 possible object pairs. This correlation reflects the extent to which to objects share a similar naming pattern within one language group.

The name similarity matrices enable us to compare naming patterns across languages since these object × object matrices can be correlated over language groups to compare the extent to which these two language groups correspond in the pairs of objects that have similar name distributions. Differences in naming patterns of French and Dutch participants are demonstrated by the imperfect correlations between the naming distributions of French and Dutch speaking monolinguals. This correlation was 0.63 for the data gathered by Ameel et al. (2005) and 0.69 in the replication data. Although there is a substantial agreement in the name similarities between both languages, these correlations indicate that French and Dutch speaking monolinguals show distinct differences in their naming pattern, both in the original data and in the replication study.

Reliability of the naming data was evaluated by applying the split-half method, followed by the Spearman–Brown correction. The reliability of the naming data was 0.96 for the bilingual participants in both French and Dutch and for the monolingual Dutch speaking participants. The naming data of the French speaking monolinguals reached a reliability of 0.95.

### Naming in Bilinguals: Testing the Hypothesis of Two Separate Naming Patterns

In order to discriminate between the one-pattern and the two-pattern hypothesis, the study of Ameel et al. (2005) presented correlational analyses and ANOVAs. Ameel et al. (2005) found a partially merged naming pattern in bilinguals. The results of the original study matched with a moderate version of the one-pattern hypothesis.

#### Group Level Analysis

**FIGURE 3:**
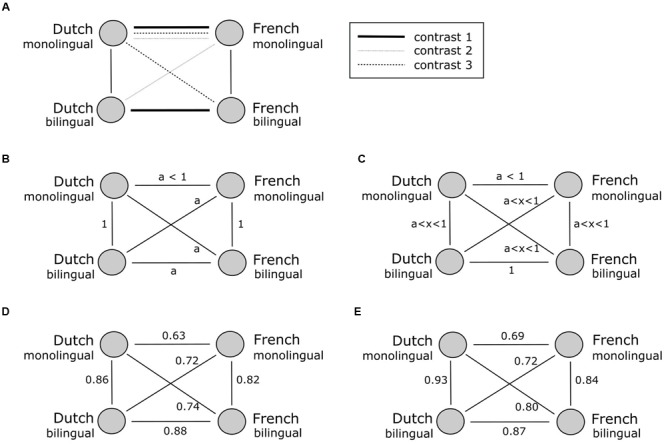
**Schematic representation of the correlation patterns between the naming similarities of each group**. The upper panel **(A)** represents the tested correlation contrasts. The middle panel represents the predicted correlation patterns corresponding with the two-pattern hypothesis **(B)** and the one-pattern hypothesis **(C)**. The lower panel **(D,E)** represents the correlation patterns of the original study **(D)** and the replication study **(E)**, respectively.

Following the original study of Ameel et al. (2005), we calculated correlations between the name similarity matrices of every language group (French monolingual, Dutch monolingual, French bilingual, and Dutch bilingual) represented in **Figure 3**. The circles correspond to the name similarities of groups speaking a specific language (Dutch-French) and of a particular linguistic status (monolingual-bilingual), the lines between the circles express the correlation between the naming patterns. The correlation between the Dutch and French bilingual naming patterns represents a within group comparison and not a within subject comparison, since the correlation is based on aggregated data.

In Ameel et al. (2005), correlation contrasts were applied to discriminate between the one- and the two-pattern hypothesis. The correlation contrasts evaluate whether there is a significant difference between two correlations. The three contrasts that were evaluated are illustrated in **Figure 3A**.

First, it was evaluated whether the correlation between the bilingual naming patterns in Dutch and French differs from the correlation between monolingual Dutch and monolingual French (*a*). The null hypothesis of this contrast corresponds to the claim of the two-pattern hypothesis that bilinguals maintain a separate naming pattern for both their languages. Ameel et al. (2005) found for both object sets that the correlation (0.88) between the two naming patterns of the bilinguals is significantly larger than *a* (0.63), *Z* = 22.98, *p* < 0.0001. We replicated this finding with a correlation of 0.87 between the naming patterns of the bilinguals that is significantly larger than *a* (0.69), *Z* = 46.45, *p* < 0.0001. We thus confirm the rejection of the two-pattern hypothesis.

Two additional correlation contrasts were tested to provide an indirect measure of the influence of one language over the other and vice versa. These contrasts are represented by the second and the third contrast in **Figure 3A**. These contrasts assume that the correlations between monolinguals in one language and bilinguals in the other language are equal to the correlation of monolinguals in both languages (*a*). This pattern is predicted by the two-pattern hypothesis and poses that there is no influence between a bilingual’s both languages. Both the second and the third contrast were found to be statistically significant. The original study reported *t* = 9.39, *p* < 0.0005 and *t* = 8.54, *p* < 0.0005 for the second and third contrast, respectively. We replicated this finding with *t* = 43.95, *p* < 0.0005 and *t* = 15.96, *p* < 0.0005 for, respectively, the second and the third contrast. Both study results reject the two-pattern hypothesis and comply with the one-pattern hypothesis. We thus confirm that the correlations between the monolingual groups with the other language of the bilingual group are significantly higher than the correlations between both monolingual language groups (*a*). This reflects the assumptions made by the strong one-pattern hypothesis and assumes direct or indirect interactions between the two languages of a bilingual. However, like the original data, these results also suggest that the strong version of the one-pattern hypothesis has to be refined since the correlation of the bilingual naming patterns does not equal 1.

#### Individual-Level Analysis

In a group-level analysis, meaningful differences between individuals or groups of individuals can be averaged out since the similarity matrices are calculated based on aggregated data. For this purpose, Ameel et al. (2005) analyzed the data on an individual level as well. To this end, they constructed an object × object matrix for every participant separately. Each cell in a participant’s matrix contains either 0 or 1, 1 indicating that a pair of objects is given the same name by a certain participant, 0 indicating that a different name is given.

**FIGURE 4:**
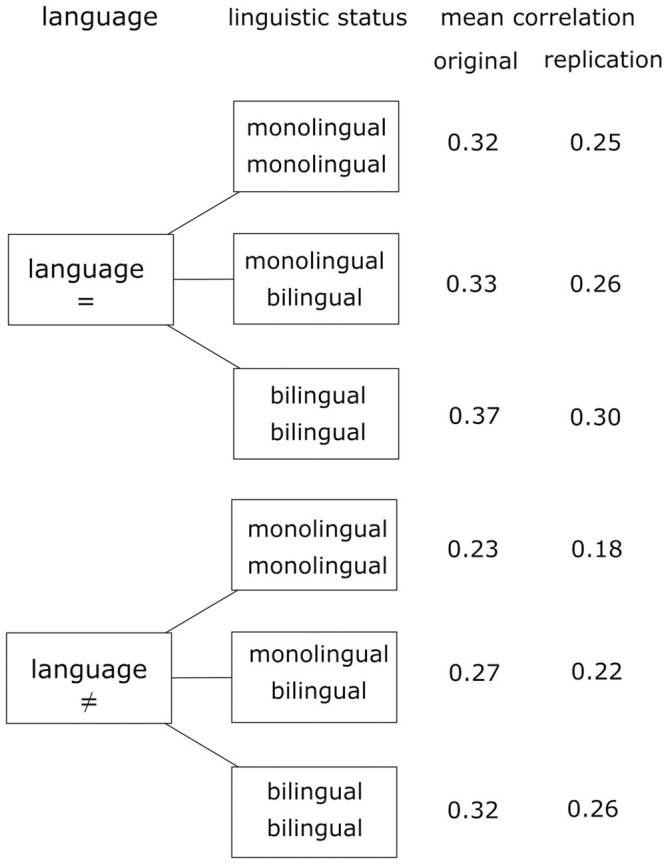
**Schematic representation of the study design**.

The replication study includes naming data of 32 monolingual Dutch speaking participants, 30 monolingual French speaking participants, and 30 bilinguals in Dutch and French. All possible pairwise between-subject correlations of naming patterns were calculated, resulting in a total of 7351 correlations. The within participant correlations were not taken into account, meaning that correlations between the naming patterns of the same bilingual performing the task in Dutch and French were left out of the analysis. To allow for a straightforward comparison with the original data, the correlations were re-analyzed with a 2^∗^3 factorial design taking only into account the factors language (with two levels, same or different language) and linguistic status (with three levels, mono-mono, bi-bi, and mono-bi). In order to normalize the sampling distribution of the correlations, they were *Z*-transformed with *Z* = 0.5^∗^ln[(1 + *r*)/(1 -*r*)]. These *Z*-transformations were analyzed using ANOVA following the original study of Ameel et al. (2005). **Figure 4** represents the new design of the study, without the within-person correlations.

Ameel et al. (2005) found an interaction of language and linguistic status, with a stronger language effect for monolinguals than for bilinguals. The presence of a language effect for bilinguals was consistent with a moderate version of the one-pattern hypothesis. The two-pattern hypothesis predicts an identical naming pattern for monolinguals and bilinguals in the same language. The predicted pattern corresponding with this hypothesis yields a main effect of language without an interaction effect of language and linguistic status. In contrast, the strong version of the one-pattern hypothesis predicts a main effect of language as well. However, according to the latter scenario the language effect only exists for monolinguals and is absent for bilinguals, since the strong version of the one-pattern hypothesis predicts a fully merged naming pattern.

We replicated the pattern found by Ameel et al. (2005) and thus confirmed the mild version of the two-pattern hypothesis. The results of the ANOVA confirmed the conclusions that were derived from the correlational group-level analysis. The two main effects – language and linguistic status – were both significant. The significant effect of language indicated that the correspondence between two naming tasks in the same language was higher than the correspondence between two naming tasks in a different language with respectively *F*(1, 6079) = 503.06; *p* < 0.0001 and *F*(1, 7349) = 482.54; *p* < 0.0001 for the original and the replication data.

Further, a significant main effect for linguistic status was found in both the original and the replication data with, respectively, *F*(2, 6079) = 172.65; *p* < 0.0001 and *F*(2, 7349) = 261.59; *p* < 0.0001. Regarding linguistic status, additional contrasts showed that both the original and the replication study found that the correlation between two bilinguals is significantly larger than the correlation between a bilingual and a monolingual with, respectively, *F*(1, 6079) = 63.44; *p* < 0.0001 and *F*(1, 7349) = 102.44; *p* < 0.0001, for the original and the replication data. The correlation between a monolingual and a bilingual is significantly larger than the correlation between two different monolinguals with, respectively, *F*(1, 6079) = 176.45; *p* < 0.0001 and *F*(1, 7349) = 258.05; *p* < 0.0001, for the original and the replication data.

In order to further discriminate between the one-pattern hypothesis and the two-pattern hypothesis, the interaction effect of language and linguistic status is crucial. Both datasets show a significant interaction *F*(2, 6079) = 25.75; *p* < 0.0001 and *F*(2, 7349) = 17.98; *p* < 0.0001 for, respectively, the original and the replication data. In order to further evaluate the interaction effect, additional contrasts were calculated. The language effect was evaluated for two levels of linguistic status (both monolingual versus both bilingual), the level monolingual-bilingual being discarded since we are not interested in this level. The main effect of language demonstrated a higher correlation within languages versus between languages. This applies both for monolinguals and bilinguals. For monolinguals, the correlation between monolinguals speaking the same language is significantly larger than the correlation between monolinguals speaking a different language with, respectively, *F*(1, 6079) = 364.70; *p* < 0.0001 and *F*(1, 7349) = 272.743, *p* < 0.0001 for the original and the replication study. For bilinguals, the correlation between two bilinguals in the same language is larger than the correlation for two bilinguals in a different language with, respectively, *F*(1, 6079) = 59.43; *p* < 0.0001 and *F*(1, 7349) = 65.83; *p* < 0.0001, for the original and the replication study. In order to confirm the moderate version of the one-pattern hypothesis, the language effect for monolinguals was contrasted with the language effect for bilinguals. This comparison proves to be significant for both studies with, respectively, *F*(1, 6079) = 36.34 and *F*(1, 7349) = 31.08; *p* < 0.0001 for the original and the replication study, indicating that the language effect for monolinguals is significantly larger than the language effect for bilinguals. The latter finding is consistent with the moderate version of the one-pattern hypothesis.

**FIGURE 5:**
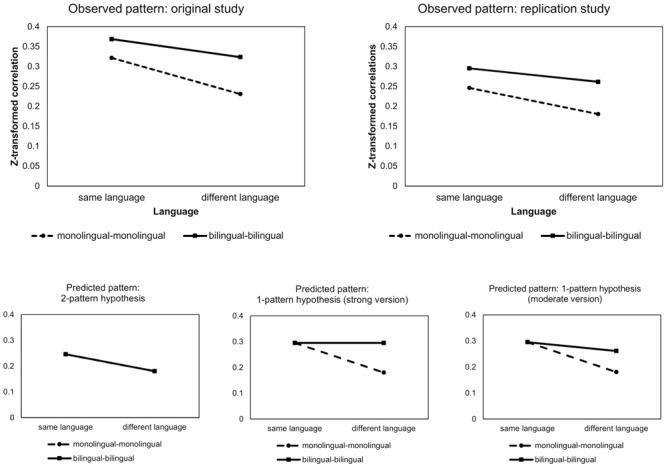
**Interaction effect between language and linguistic status**. The upper panel shows the observed pattern for the original and replication study. The lower panel shows the predicted patterns in case of the two-pattern hypotheses, the one-pattern hypothesis, and the moderate version of the one-pattern hypothesis.

In **Figure 5**, the observed patterns are depicted in a schematic overview. The upper panel contains the pattern found in the original data and the replication data. Although the average correlations for the replication data (based on the larger stimulus set) are slightly lower than the correlations in the original study, it is clear that the replication study results in the same pattern. As demonstrated by the ANOVA-analysis both monolinguals and bilinguals demonstrate an effect of language, that is, correlations are higher in the same language compared to correlations between languages. However, the language effect for bilinguals is smaller than the effect for monolinguals. The better correspondence in different languages for bilinguals is an indication for convergence of the naming pattern in both languages. This convergence is, however, only partial since there is still a small effect of language present in bilinguals. The patterns in this replication study correspond to a moderate version of the one-pattern hypothesis, supporting the idea of a partially merged naming pattern in bilinguals. The predicted patterns corresponding to the two-pattern hypotheses, the one-pattern hypothesis, and the moderate version of the one-pattern hypothesis can be found in the lower panel of **Figure 5**.

### Convergence in a Geometrical Space

After successfully replicating the findings of Ameel et al. (2005) concerning the partial convergence of naming patterns in bilinguals, we also replicated a study described in Ameel et al. (2009) shedding light on the nature of this convergence. To this end, the linguistic categories were presented in a multidimensional geometrical space in which every category exemplar is represented by a vector of M coordinates, each vector reflecting one of the M underlying psychological dimensions (Smith and Minda, 1998; Smits et al., 2002). The category prototype can be represented as a centroid point in this geometrical representation (Rosch and Mervis, 1975; Hampton, 1979).

Ameel et al. (2009) found that centers of roughly corresponding categories in Dutch and French for bilinguals were located closer to each other than the centers of corresponding categories for monolinguals. Furthermore, the bilingual categories were situated somewhere in between the monolingual categories for French and Dutch monolinguals, because the overlap in corresponding categories for bilinguals drives the two centers toward each other along the lines that connect the monolingual category centers. Furthermore, they found that bilingual categories were situated significantly closer to each other, regardless of whether the centroids were calculated in a boundary-dependent or a boundary-independent way. In order to calculate the boundary-dependent location of a category center, Ameel et al. (2009) calculated a weighted average of the coordinates of the object set, with the weights being the name frequency for that category. In this boundary-dependent way of calculation, all items contribute to the location of the category center, the boundary exemplars included. A boundary-independent location of the category center was calculated by using the weighted median, with the weights again being the name frequency for that category. The median value is not influenced by outliers and hence produces a boundary-independent central tendency measure. The fact that convergence was shown in both category boundaries and centers is an indication that the source of the overall convergence lies in both places. Category centers are mostly determined by high-frequency items, unlike category boundaries that are mainly formed by low-frequency items. Category boundaries could also be more strongly determined by language specific idiosyncrasies (Malt et al., 1999). The data suggested that bilinguals cannot keep the exemplars of each language separate since there is manifestation of convergence even at the level of category centers. Supplementary Figure 1 illustrates the category centers of bilinguals and monolinguals for both languages for the replication data.

A common underlying representation reflecting the similarity between the objects was the starting point to compare the locations of category centers for the different language groups in the current study. Unlike the research of Ameel et al. (2009), the sorting task was only performed by monolingual Dutch speaking participants. For every participant an individual similarity matrix was calculated based on how the stimuli were grouped together. If two stimuli were sorted into the same pile this was indicated with 1 in the similarity matrix, if two stimuli were sorted in separate piles this was indicated with 0. Since participants were allowed to cluster piles, some additional calculations were done depending on the number of piles within one cluster. For example, if a participant clustered two piles, the stimulus pairs within one pile still received 1, but stimulus pairs within the same cluster, but not sharing the same pile, received 0.5. In the case three piles were clustered again, stimulus pairs in the same pile received 1, stimulus pairs within the cluster but not sharing the same pile received 0.33. Therefore, the number of piles within a cluster was taken into account as well. The reliability of the sorting data was estimated using the split-half technique followed by the Spearman-Brown formula. The average pairwise similarity for 10,000 random splits of the sorting data reached 0.92 and thus proves to be highly reliable.

**FIGURE 6:**
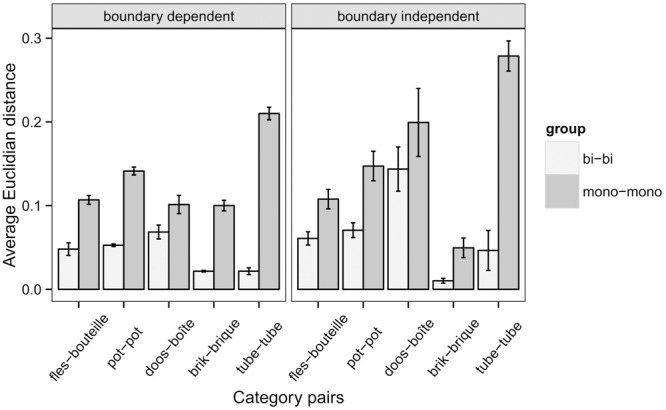
**Average Euclidian distance between roughly equivalent category pairs in Dutch and French for bilinguals and monolinguals over 2–7 dimensions with standard error of the mean**.

Based on the naming data, the positions of the centers for different category names were determined for each of the language groups in multidimensional scaling (MDS) representations. This procedure was done for the roughly corresponding Dutch-French categories *fles*-*bouteille, pot*-*pot, doos*-*boîte, brik*-*brique*, and *tube*-*tube*, and repeated for 2–7 dimensions. **Figure 6** displays the average Euclidian distances for monolinguals and bilinguals between roughly equivalent category pairs in Dutch and French over 2–7 dimensions. Like in the research by Ameel et al. (2009), the category centers for bilinguals in French and Dutch are consistently closer to each other than the category centers for monolinguals in French and Dutch.

A two-sample *t*-test demonstrated that the average distance in category centers between bilinguals is significantly smaller than the average distance between roughly corresponding centers for monolinguals. This is the case for the boundary dependent measure [*t*(29) = 8.90, *p* < 0.0001] and for the boundary independent measure [*t*(29) = 6.01, *p* < 0.0001]. Convergence takes place at both category boundaries and category centers confirming the findings of Ameel et al. (2009).

**FIGURE 7:**
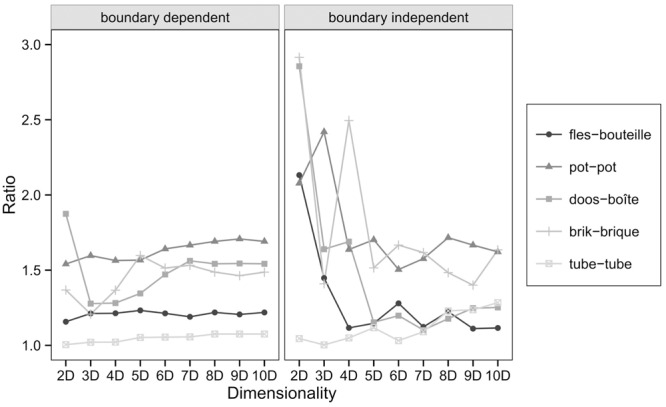
**Ratios of the indirect distance to the direct distance between monolingual category centers**.

To answer the question whether the bilingual categories consistently lie in between the monolingual categories, the ratio of the indirect distance to the direct distance for each monolingual category pair was calculated. The indirect distance consists of the summed distance from the monolingual Dutch to the bilingual Dutch center, from bilingual Dutch to bilingual French and bilingual French to monolingual French. If the bilingual categories are indeed situated in between the monolingual category centers, then the ratio of the indirect distance to the direct distance should be approximately one. Ameel et al. (2009) reported that this was the case indeed. **Figure 7** displays the ratios of the indirect distance to the direct distance of the replication study. However, unlike the original study, this is only consistently the case for the category pair *tube-tube* regardless of the dimension or whether the centers were calculated in a boundary dependent or boundary independent way.

The replication study does not confirm the finding that bilingual centers are consistently situated in between monolingual category centers in the dimensionalities under study. The inconsistent ratios in lower dimensionalities could indicate that a low dimensional representation of this stimulus set, which, with 192 stimuli is considerably larger than the one used in Ameel et al. (2009), does not suffice. Results in lower dimensionalities would therefore be distorted and not reliable. If dimensionality is the problem, one would expect that the ratios decrease in higher dimensionalities. However, as can be seen in **Figure 7**, the ratios do not decrease systematically with an increasing dimensionality (Dimension 8–10 were added to evaluate this possibility).

Another possible explanation why we do not observe the category centers of the bilingual participants in the middle of the monolingual centers, could be the rather short distance between the monolingual category centers. Even a small deviation of one of the bilingual category centers from the straight line connecting the monolingual centers causes an increased ratio of the indirect distance to the direct distance. If we take into account the distances between the monolingual category centers in the interpretation of the ratios in **Figure 7**, there is a significant relation between the distance between monolingual centers and the ratios of the indirect distance to the direct distance between monolingual category centers both for boundary dependent and boundary independent calculations. Spearman rank order correlations over 2–7 dimensions show that the larger the distance between monolingual category centers, the smaller the ratio both for boundary dependent (*r*_s_ = -0.60, *p* < 0.001) and boundary independent (*r*_s_ = -0.73, *p* < 0.001) calculations. This is an indication that, for the categories with a small difference in distance between monolingual category centers, the ratios of the indirect distances to the direct distances are not reliable and therefore not interpretable.

## Conclusion and Discussion

This conceptual replication study offers confirmatory evidence for a converged naming pattern in adult bilinguals as demonstrated by Ameel et al. (2009, 2005). The convergence in the bilingual lexicon reliably manifested itself in a series of experiments conducted by Ameel et al. (2009, 2005) and in addition we succeeded in replicating these experiments.

We successfully replicated the group-level analysis with correlation contrasts. Both the original study and the replication study reject the two-pattern hypothesis and comply with the one-pattern hypothesis. Furthermore, both studies are in favor of a moderate version of the one-pattern hypothesis since the correlation of the bilingual naming patterns does not equal 1. Individual level analysis using ANOVA further confirmed the earlier findings, since correlations were found to be higher in the same language compared to correlations between languages. However, this language effect is smaller for bilinguals than for monolinguals. The better correspondence in different languages for bilinguals is once more an indication for convergence of the naming patterns in both a bilingual’s languages. Moreover, this convergence is again found to be only partial, considering the small effect of language present in bilinguals. The correlation patterns in this replication study, like in the original study of Ameel et al. (2005), correspond to a moderate version of the 1-pattern hypothesis supporting the idea of a partially merged naming pattern in bilinguals.

More evidence for converged naming patterns was found in a representation in a multidimensional space and the finding of shorter distances between bilingual category centers than monolingual category centers in a multidimensional space. These findings show that convergence takes place at both category boundaries and category centers. However, the ratios of the indirect distance to the direct distance for each monolingual category pair are not found to be close to one. A possible explanation why we did not succeed in replicating this finding lies in the rather small distances between monolingual category centers. These small distances could boost small deviations from the straight line connecting monolingual category centers and could cause an increased ratio, which renders these ratios unreliable and uninterpretable. This possibility is supported by the Spearman rank order correlations indicating that the larger the distance between monolingual category centers, the smaller the ratio of the indirect distance to the direct distance.

Since we assume a shared underlying representation of the similarity between objects, the convergence we observe only manifests itself at the level of the naming pattern and not at the representation level. The languages under study are rather similar and some object labels are cognates (e.g., *pot, tube* or *flacon*). Intuitively, one would assume that convergence will be strongest for labels with a larger similarity in naming patterns between both languages. However, this does not mean that convergence can only manifest itself in bilinguals speaking two languages that are rather similar. A study with late Mandarin – English bilinguals shows convergence in naming patterns despite immersion at a later age, their languages being quite dissimilar and not having any cognates (Malt et al., 2015).

This pre-registered replication project provides strong evidence for the finding of convergence in the bilingual lexicon. Since we were able to replicate semantic convergence over the full series of experiments described by Ameel et al. (2005, 2009), the evidence for convergence in naming patterns is robust. Taking into account that this study is a conceptual replication is a further support for the robustness and the reliability of the semantic convergence found in the original study. Since replication is an important tool in verifying hypotheses (Schmidt, 2009) we not only confirmed the finding of a converging bilingual lexicon, but also verified the moderate version of the one-pattern hypothesis. The importance of this study does not lie only in the success of the replication. Given the current crisis of confidence in psychology, replication studies in general are important to provide evidence for the credibility of psychological research and to support earlier obtained results (Schmidt, 2009). Furthermore, all aspects of this project were pre-registered, which adds further value to the confirmatory evidence for the original findings of Ameel et al. (2005, 2009).

Although we can confirm the merged naming pattern in bilingual adults, these results do not give decisive information about semantic convergence in types of bilingualism other than simultaneous early bilinguals. Although we went through a considerable amount of effort to include balanced bilinguals, we were not able to find a perfectly balanced group of bilingual participants. This was also the case for the original study of Ameel et al. (2005) as shown by the very comparable proficiency estimates. Completely balanced bilinguals are quite rare, since there is almost always one language of which the presence is more dominant in daily life. As we can observe in our sample, only 8 out of 30 participants indicate an equal proficiency in both Dutch and French. The largest group of participants indicated dominance in Dutch (19 participants). At a group level the bilingual participants indicated a significantly higher degree of proficiency in Dutch than in French. We did indeed observe higher correlations between monolingual Dutch and bilingual Dutch, and between monolingual Dutch and bilingual French as shown in **Figure 3E**. These correlations are higher than the correlations between monolingual French and bilingual French and monolingual French and bilingual Dutch, respectively. This could very well be a reflection of the language imbalance in our group of participants and the dominance of Dutch at a group level.

In recent literature, it is suggested that there is a bidirectional influence between a bilingual’s two languages, even in late bilinguals or second language learners (Malt et al., 2015). It is suggested that this influence is driven by the extent to which a bilingual’s languages are balanced (Bylund and Athanasopoulos, 2015). If the degree to which the converged naming pattern in bilinguals inclines toward one of the monolingual naming patterns is driven by language dominance and language proficiency, the bilinguals’ higher proficiency in Dutch could explain the higher correlation between monolingual Dutch and bilingual Dutch. If we consider bilingualism to be a continuum with one end of the scale completely balanced early simultaneous bilinguals and on the other end second language learners, it could be possible to observe convergence to a higher or lower degree modeled by language proficiency in both languages in other types of bilingualism as well.

## Ethics Statement

The study was approved by the KU Leuven Social and Societal Ethics Committee. All subjects gave written informed consent.

## Author Contributions

AW gathered the data, drew all the diagrams and wrote the first draft of all sections. All authors discussed the general outline of the article and contributed with comments and revisions.

## Conflict of Interest Statement

The authors declare that the research was conducted in the absence of any commercial or financial relationships that could be construed as a potential conflict of interest.

## Footnotes

1 We calculated correlations of individual naming patterns with the average naming pattern in the mother tongue and the foreign language for all participants. None of the monolingual participants who indicated a high proficiency in the other language are outliers, not in the correlation with their mother tongue nor the correlation with their foreign language. Therefore it is not possible that these participants influence the outcome of the analyses presented below.

2 In French diminutive forms require an adjective. Since we focus on the head noun of the response it is necessary to combine diminutive and non-diminutive forms in Dutch to allow a straightforward comparison between both languages.
