# Supplementary material for: Convergence in the Bilingual Lexicon: A Pre-registered Replication of Previous Studies
Source: Front Psychol. 2017 Jan 23;7:2081. doi: 10.3389/fpsyg.2016.02081 (PMC5253376; doi:10.3389/fpsyg.2016.02081)
Supplement: Supplementary file 1 [file Data_Sheet_1.docx]

Supplementary Material

Convergence in the bilingual lexicon: A pre-registered replication study

Anne White*, Co-Author, Barbara C. Malt, Gert Storms

*** Correspondence:** Corresponding Author: anne.white@kuleuven.be

**Supplementary Table 1.** Linguistic categories for the bottles set for Dutch and French speaking monolinguals.

| Dutch bottles | N | French bottles | N | Dutch composition |
| --- | --- | --- | --- | --- |
| fles | 76 | bouteille | 47 | 45 flessen, 1 bus, 1 fles/bus |
| pot | 33 | pot | 34 | 22 potten, 4 flessen, 3 emmers, 2 dozen, 1 beker, 1 bokaal, 1 tube |
| doos | 16 | tube | 21 | 7 tubes, 4 flessen, 2 potten, 2 bussen, 1 brik, 1 doos, 1 fles/tube, 1 roller, 1 zak |
| bus | 14 | flacon | 19 | 15 flessen, 3 potten, 1 staal |
| brik | 11 | boîte | 19 | 13 dozen, 3 blikken, 2 brikken, 1 vloot |
| tube | 9 | spray | 12 | 9 bussen, 2 flessen, 1 fles/bus |
| blik | 5 | berlingot | 6 | 4 brikken, 2 zakken |
| zak | 4 | bidon | 4 | 2 bidons, 1 bus/blik, 1 fles |
| fles/bus | 3 | gourde | 3 | 2 bussen, 1 zak |
| emmer | 3 | biberon | 2 | 2 flessen |
| bidon | 2 | canette | 2 | 2 blikken |
| beker | 2 | doseur | 2 | 1 beker, 1 pot |
| vat | 1 | fiole | 2 | 1 buis, 1 fles/kolf |
| fles/tube | 1 | pot/boîte | 2 | 2 potten |
| kan | 1 | boîte/carton | 1 | 1 brik |
| bokaal | 1 | bonbonne | 1 | 1 fles |
| fles/kolf | 1 | bouteille/biberon | 1 | 1 fles |
| roller | 1 | bouteille/bidon | 1 | 1 fles |
| vloot | 1 | bouteille/tube | 1 | 1 fles |
| staal | 1 | brique | 1 | brik |
| mand | 1 | brique/carton | 1 | brik |
| thermos | 1 | casserole | 1 | 1 pot |
| bus/blik | 1 | cubi | 1 | 1 brik |
| buis | 1 | moulin | 1 | 1 molen |
| vaas | 1 | panier | 1 | 1 mand |
| molen | 1 | pot/bidon | 1 | 1 kan |
|  |  | pot/tube | 1 | 1 fles/bus |
|  |  | salière | 1 | 1 vat |
|  |  | théière | 1 | 1 pot |
|  |  | thermos | 1 | 1 thermos |
|  |  | vase | 1 | 1 vaas |

**Supplementary Figure 1.** Two-dimensional MDS representation of the category centers for the four different language groups. The upper panel shows the boundary dependent category centers, the lower panel shows the boundary independent category centers.
